# Assessment of autoregressive integrated moving average (ARIMA), generalized linear autoregressive moving average (GLARMA), and random forest (RF) time series regression models for predicting influenza A virus frequency in swine in Ontario, Canada

**DOI:** 10.1371/journal.pone.0198313

**Published:** 2018-06-01

**Authors:** Tatiana Petukhova, Davor Ojkic, Beverly McEwen, Rob Deardon, Zvonimir Poljak

**Affiliations:** 1 Department of Population Medicine, Ontario Veterinary College, University of Guelph, Guelph, ON, Canada; 2 Animal Health Laboratory, Laboratory Services Division, University of Guelph, Guelph, ON, Canada; 3 Department of Production Animal Health, University of Calgary, Calgary, AB, Canada; 4 Department of Mathematics and Statistics, University of Calgary, Calgary, AB, Canada; Columbia University, UNITED STATES

## Abstract

Influenza A virus commonly circulating in swine (IAV-S) is characterized by large genetic and antigenic diversity and, thus, improvements in different aspects of IAV-S surveillance are needed to achieve desirable goals of surveillance such as to establish the capacity to forecast with the greatest accuracy the number of influenza cases likely to arise. Advancements in modeling approaches provide the opportunity to use different models for surveillance. However, in order to make improvements in surveillance, it is necessary to assess the predictive ability of such models. This study compares the sensitivity and predictive accuracy of the autoregressive integrated moving average (ARIMA) model, the generalized linear autoregressive moving average (GLARMA) model, and the random forest (RF) model with respect to the frequency of influenza A virus (IAV) in Ontario swine. Diagnostic data on IAV submissions in Ontario swine between 2007 and 2015 were obtained from the Animal Health Laboratory (University of Guelph, Guelph, ON, Canada). Each modeling approach was examined for predictive accuracy, evaluated by the root mean square error, the normalized root mean square error, and the model’s ability to anticipate increases and decreases in disease frequency. Likewise, we verified the magnitude of improvement offered by the ARIMA, GLARMA and RF models over a seasonal-naïve method. Using the diagnostic submissions, the occurrence of seasonality and the long-term trend in IAV infections were also investigated. The RF model had the smallest root mean square error in the prospective analysis and tended to predict increases in the number of diagnostic submissions and positive virological submissions at weekly and monthly intervals with a higher degree of sensitivity than the ARIMA and GLARMA models. The number of weekly positive virological submissions is significantly higher in the fall calendar season compared to the summer calendar season. Positive counts at weekly and monthly intervals demonstrated a significant increasing trend. Overall, this study shows that the RF model offers enhanced prediction ability over the ARIMA and GLARMA time series models for predicting the frequency of IAV infections in diagnostic submissions.

## Introduction

Influenza A virus (IAV) circulates in swine populations worldwide and has recently been characterized by the continuous emergence of novel viral recombinants and variants in some regions [1–3]. Coupled with the complex demographics of swine populations and their high birth and replacement rate, such viral diversity could result in increased incidence of influenza infection and present a challenge for the development of infection and disease control strategies in animal populations. This could also cause some concerns from the public health perspective, similar to those caused by the spill-over infections from swine to people observed in 2012 in the US [4]. Thus, the development of new surveillance methods for IAV has its merits from multiple perspectives. Among different goals of surveillance, an important objective is the establishment of the capacity to forecast with the greatest accuracy the number of influenza cases likely to arise. Such an objective could be accomplished on the basis of statistical data-driven models, and is important whether the infection occurs as a major epidemic of a single strain, during the endemic state characterized by the continuous circulation of existing strains, or under the limited emergence of novel strains. Such an approach to forecasting could represent the basis for planning resource allocation by both animal and public health authorities. Of course, a reliably high forecasting accuracy would be key.

Diagnostic submissions for IAV from swine populations in Ontario, Canada, repeatedly peak in January and April [5]. For diseases that show recurrent seasonal patterns or occur in cyclic patterns, time series models are the most widely used statistical models by health researchers for forecasting [6]. Time series forecasting is commonly performed using autoregressive integrated moving average (ARIMA) models [6] that can accommodate both trend and seasonal variations. ARIMA models are typically selected by maximizing some measure of predictive accuracy. However, a drawback of ARIMA models is that they assume a Gaussian distribution of the response. Given count data, a Box-Cox transformation of counts using either a logarithmic or power transformation may yield approximately Gaussian-distributed data. Nevertheless, Gaussian modeling with transformed data may result in an inaccurate predictive distribution.

Another approach developed by Davis et al. [7] uses generalized linear autoregressive moving average (GLARMA) models. These models accommodate time series of counts that are assumed to follow a Poisson distribution. Recently, Dunsmuir et al. [8] developed an automated algorithm that provides for model identification within a given class of models and an assessment of model adequacy in regression modeling of count time series that follow Poisson, negative binomial or binomial distributions.

An additional, alternative approach for modeling count data is to use random forest (RF) models as developed by Breiman [9]. RF models offer a rule-based methodological approach that recursively partition data, creating regression trees. RF models have been successfully applied in many fields, including public health studies. Studies by Cootes et al. [10] and Kane et al. [11], among others, suggest this modeling approach provides computational efficiency and high predictive accuracy.

Despite the fact that ARIMA, GLARMA, and RF models have been used in several studies, these approaches have never been applied to time series data for IAV surveillance or any other pathogens in swine populations. Furthermore, recent studies highlight the fact that swine have the highest rate of emergence of new viral infectious agents and, therefore, enhanced surveillance and comprehensive assessment are needed [12]. Thus, the objective of this study is to compare the performance of ARIMA, GLARMA, and RF models with respect to predicting the frequency of IAV in diagnostic submissions from swine populations in Ontario. Our main interest was to identify a model that would predict increases in the number of diagnostic submissions and positive virological submissions with a high degree of sensitivity, using data based on diagnostic submissions to the Animal Health Laboratory (AHL; University of Guelph, Guelph, Ontario, Canada). We were also interested in investigating the occurrence of seasonality and the long-term trend of IAV infections by applying time series and recursive partitioning modeling approaches to the same data from the AHL.

## Materials and methods

### Data processing

**Fig 1:**
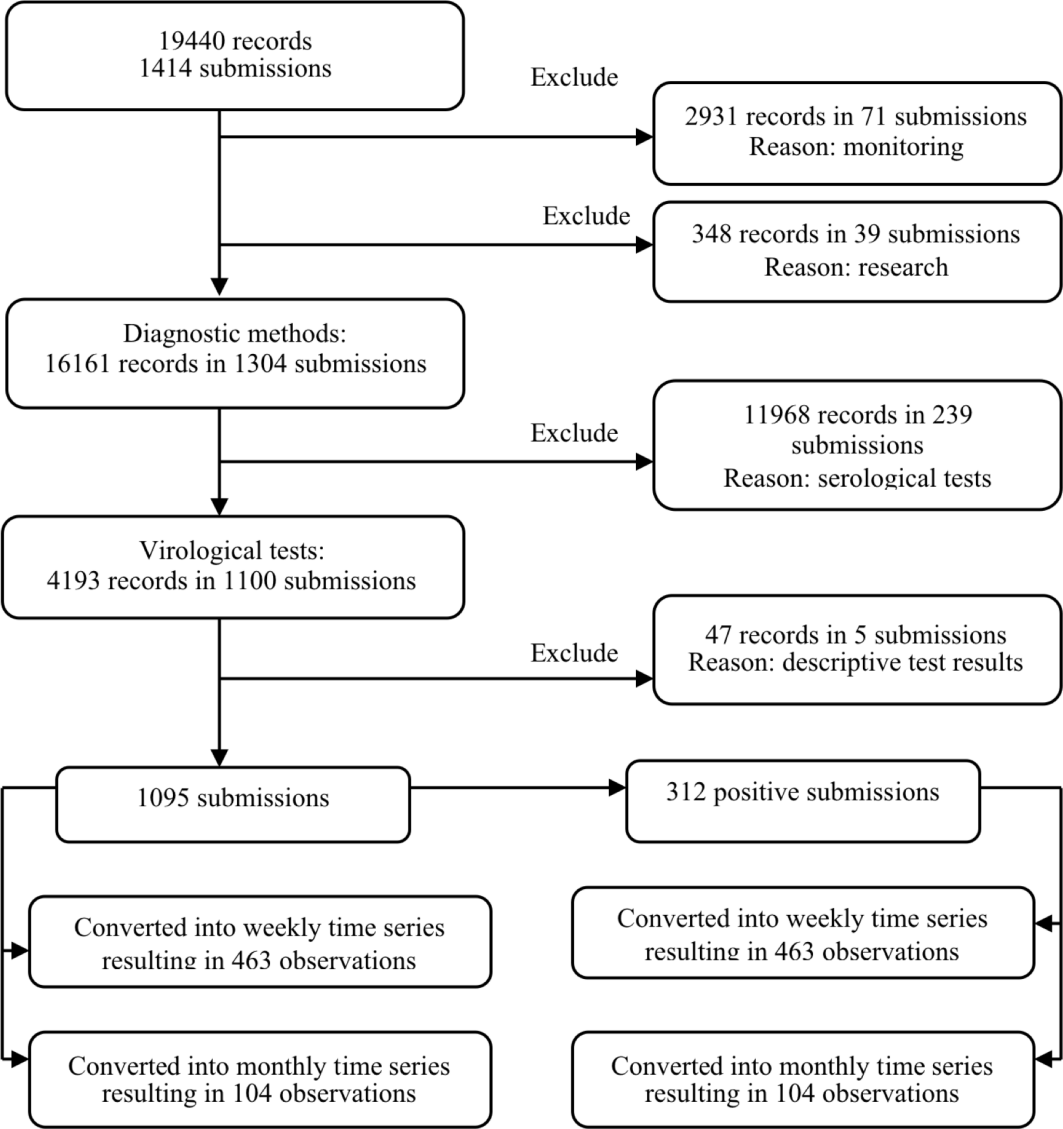
Graphical illustration of data processing procedure. Data were obtained from records of swine submissions supplied from farms in Ontario, Canada, to the Animal Health Laboratory over the period from May 2007 to December 2015.

Our data set contain the test-level records from porcine submissions that were voluntarily supplied from Ontario swine farms between May 2007 and December 2015 at the largest animal health diagnostic laboratory (AHL) in Ontario. We processed the data and extracted relevant information for the analysis, as shown in Fig 1.

Each diagnostic submission contained one or more samples, and all samples were tested for IAV for research, monitoring, or diagnostic purposes. The test results for research and monitoring were excluded from the analysis based on the rationale that they might not represent actual clinical influenza disease in a herd. The diagnostic test results indicated if the samples were tested using serological and/or virological methods. We disregarded the serological findings since a serological diagnosis is based on the detection of antibodies, and could be a consequence of vaccination or historical exposure at an unknown point in time. Virological tests were from procedures such as real-time reverse transcription polymerase chain reaction (rtPCR), immunohistochemistry, or virus isolation. These techniques were applied to either different samples or the same sample within a submission. Test results interpreted as “inconclusive”, “suspicious”, or “weak positive” were considered negative. When a categorical result (positive/negative) was not declared for quantitative real-time rtPCR test results, we used a cycle threshold (Ct) to declare a test result. Ct is the (amplification) cycle number when fluorescence increases above the background level. The reagents employed were manufactured by Life Technologies, and we used their recommended Ct of 36 as a positive cut off value; that is, Ct ≤ 36 was used to declare a positive test. Any test that indicated a positive result for influenza A virus was considered a positive individual virological test, and a submission with at least one positive individual virological test was considered a positive submission. Test results reported descriptively were excluded from the analysis. The number of daily submissions, and the number of daily positive diagnostic submissions at the herd level, were aggregated into monthly and weekly intervals with variables corresponding to the date of the beginning of the week or month, resulting in four individual historical datasets. A week was considered to run from Monday to Sunday and each study year included 52 weeks. The 53^rd^ week for years 2007 and 2012 was omitted to ensure the same number of weeks in each study year and enable simple conversion of the outcome measures into comparable time series. The time series of the number of diagnostic submissions, and positive virological submissions at weekly and monthly intervals were analyzed individually.

### Data

Each historical data set contains information on the outcome measures and variables that were included in the analysis. The outcome measures of interest were the time series of: (i) the number of diagnostic submissions per week, (ii) the number of diagnostic submissions per month, (iii) the number of positive virological submissions per week, and (iv) the number of positive virological submissions per month. A detailed description of the explanatory variables used is given under each model description.

### Statistical methods

A time series decomposition was performed on the four historical time series. Using the results from the decomposition, ARIMA, GLARMA and RF models were built to assess and predict the frequency of IAV in the swine population in Ontario. The predictive accuracy of each modeling approach was evaluated via the root mean square error (RMSE) and the normalized root mean square error (NRMSE). We also assessed the models’ ability to anticipate increases and decreases in the number of diagnostic submissions and positive virological submissions at weekly and monthly intervals.

We implemented a seasonal-naïve method based on weekly/monthly averages over past years for each historical time series and it was used as a benchmark comparison for the ARIMA, GLARMA and RF models. The seasonal-naïve method was assessed in the same way as the other models.

The statistical analyses were performed using R version 3.3.1 [13] with the significance level set at P < 0.05. An enhanced description of each methodology is contained in the Supplementary Material (S1 File). The computation methods for the RMSE and NRMSE are also elaborated upon in the Supplementary Material (S1 File).

#### Time series decomposition

Before applying the time series techniques, the four outcome measures were investigated for temporal autocorrelation in the residuals using the Durbin-Watson test. Under this test, the null hypothesis is that the residuals are serially uncorrelated, and this is tested against the alternative hypothesis that they follow a first-order autoregressive process. The value of the test statistics observed suggested a pattern of positive serial correlation for each series (d < 2). This was also confirmed by examining the time series graphically. Applying a filtering procedure, the time series were decomposed into trend, season, and remainder components using the STLPLUS function [14]. The procedure is based on a local regression smoother. The naïve smoothing parameter, *n_s_*, was set to 19 lags based on the seasonal-diagnostic plots. The value of trend window, *n_t_*, was calculated considering the frequency of the time series and the seasonal smoothing parameter and assessed with trend-diagnostic plots [14]. For the weekly time series, *n*_t.week_ was set to 87 lags; for the monthly time series, *n*_t.month_ was set to 21 lags. The robust STLPLUS estimation procedure was used based on the seasonal-diagnostic and trend-diagnostic plots. For the robust procedure, the number of inner iterations used was 2 and the number of outer iterations used was 5, which provided convergence of the procedure.

#### ARIMA

We first considered the representation of the observed time series via an ARIMA model. Identifying and fitting an ARIMA model can be quite complex and time consuming as it can have a large number of parameters. Therefore, the model estimation procedure was performed using the stepwise automatic algorithm with the AUTO.ARIMA function in R. The best of all possible models was selected according to Akaike’s Information Criterion (AIC) [15]. A Box-Cox transformation was used to help satisfy the ARIMA assumptions.

#### GLARMA

Because the time series of the number of diagnostic submissions and positive virological submissions per week and per month consist of counts, it is natural to model them using GLARMA models. The GLARMA modeling process was performed on the four historical count time series using the R package GLARMA [8]. The explanatory variables used were the linear trend and the season. The trend represents an increase by one unit over the entire study period and was centered at the mid-point of the study period. The season effect was introduced as a categorical variable with either four levels (winter, spring, summer, and autumn, with summer used as the reference level) for the weekly historical count time series or 12 levels (12 months of the year, with August used as the reference level) for the monthly historical count time series. The ARMA components were selected based on the estimated autocorrelation and partial autocorrelation functions using the residuals from the generalized linear model regression. The best model was selected based on the Wald test, the likelihood ratio test, and the AIC; here these measures were always in agreement. The response distribution for each time series was selected depending on the estimated value of the shape parameter: if the parameter was significant, a negative binomial distribution was used; otherwise, a Poisson distribution was used. The validity of the assumed distribution was examined via the probability integral transformation.

#### Random forests

Finally, random forest regression [9] was used to analyze the four time series. Regression was performed with the R randomForest package [16]. Explanatory variables included were the linear trend and the season (as for the GLARMA model), as well as the count time series up to five lags. The importance of each variable was calculated. The performance of RF-based regression was evaluated and optimized for the smallest error estimate via 10-fold cross-validation (CV) and the “out-of-bag” (OOB) error.

### Retrospective analysis

A retrospective analysis was performed on each historical time series for the period from May 2007 to December 2015. In this approach, the ARIMA, GLARMA, and RF models were built to assess the effect of seasonality and the long-term trend of IAV infections on the number of diagnostic submissions and positive virological submissions at weekly and monthly intervals.

### Simulated prospective analysis

A simulated prospective analysis was performed on each historical time series to compare the performance of the ARIMA, GLARMA and RF models. The simulations started by training a model on the first 44 weeks (or months) of data. The process proceeded by iteratively adding a successive week (or month), retraining the model using the updated data, and predicting the number of submissions or positive submissions, excluding the training period. This process is known as “forecast evaluation with a rolling origin” [17]. The ARIMA, GLARMA and RF models in the simulated prospective analysis were built in a similar fashion to the retrospective analysis. The predictions and residuals were examined graphically to verify the adequacy of different aspects of the model. We also implemented leave-one-season-out cross-validation, LOSO, where each season was successively “left out” from the training period and used for validation.

To investigate the ability of each model to predict increases and decreases in the number of diagnostic submissions and positive virological submissions, confusion matrices were constructed where predicted increases and decreases were classified into correctly (and incorrectly) identified actual increases and decreases. The accuracy for each modeling approach was calculated to determine the proportion of the total number of predictions that were correct. Sensitivity, the proportion of correctly identified increases, was also computed.

## Results

### Retrospective ARIMA, GLARMA, and RF

Overall, 1414 unique submissions from swine herds were submitted to the AHL. Of the 1304 (92.2%) diagnostic submissions, 1100 (84.4%) were tested with virological procedures (Fig 1). Of these, 1095 (99.6%) submissions, including 312 (28.5%) positive submissions, were aggregated based on the submission date to obtain the number of diagnostic submissions and positive virological submissions per week and month. In total, 463 weekly and 104 monthly observations were converted into the time series used for analysis. Total weekly diagnostic submissions ranged from 0 to 11, increasing from an average of 2.5 per week in 2007 to 3 per week in 2015. Total weekly positive virological submissions ranged from 0 to 6, increasing from an average of 0.5 per week in 2007 to 1.3 per week in 2015. Total monthly diagnostic submissions ranged from 2 to 23, increasing from an average of 10.8 per month in 2007 to 13.3 per month in 2015. Total monthly positive virological submissions ranged from 0 to 13, increasing from an average of 2 per month in 2007 to 5.8 per month in 2015.

**Fig 2:**
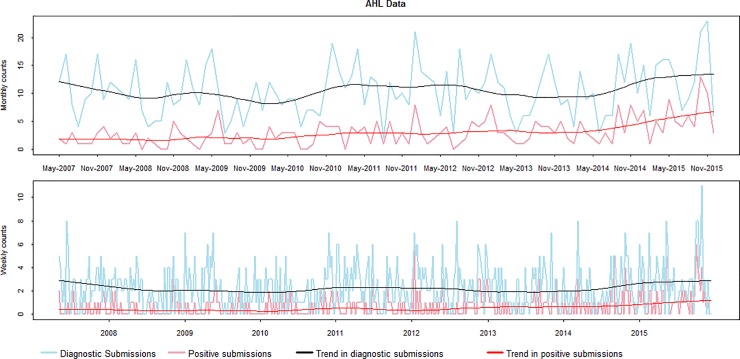
Number of diagnostic submissions and positive virological submissions for IAV per week and month. The counts were obtained from swine samples submitted to the Animal Health Laboratory in Ontario from May 2007 (week 19) to December 2015 (week 52). The original data are represented by blue lines for diagnostic submissions and by pink lines for positive virological submissions. The four time series were subjected to the decomposition, and the somewhat upward trend-cycle component in diagnostic submissions is shown in black while the slow increasing trend in positive counts is displayed in red.

The four count time series are shown in Fig 2. The series seem to exhibit seasonal fluctuations. The trend from the weekly and monthly diagnostic submissions is apparent from visual inspection and seems to behave in a cyclic manner but with some tendency to upward drift. The positive counts at weekly and monthly intervals show a slow increasing trend.

**Fig 3:**
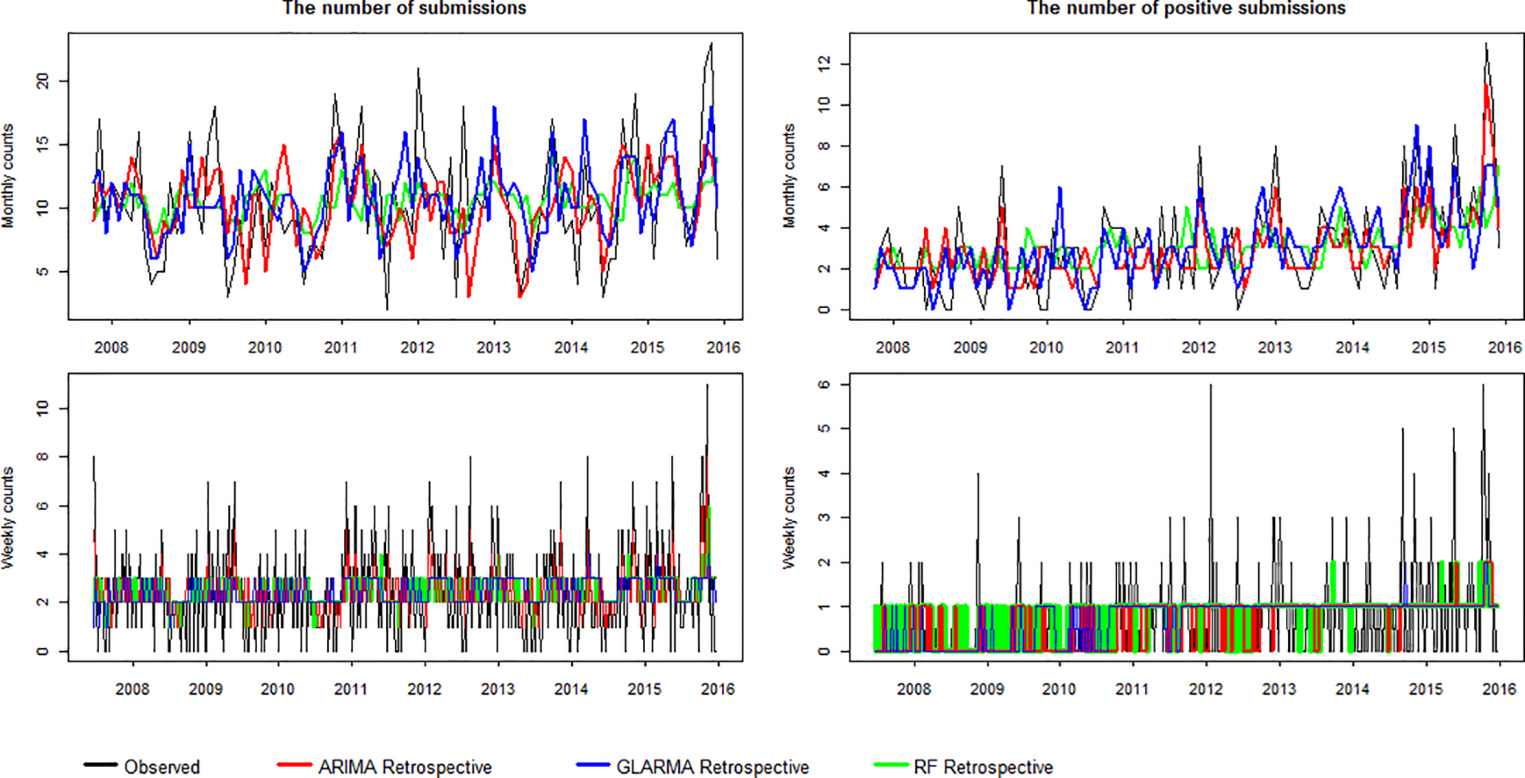
Retrospective predicted counts of weekly and monthly submissions and positive submissions for IAV. The autoregressive integrated moving average (ARIMA) is shown in red, the generalized linear autoregressive moving average (GLARMA) in blue, and the random forest (RF) in green. The actual observations are represented by black lines.

The ARIMA analysis indicated the presence of trend in the time series of the number of monthly diagnostic submissions, and the number of weekly and monthly positive virological submissions. First differencing reduced the effect of the trend. The coefficient estimates of the retrospective ARIMA components are provided in S1 Table in the Supporting Materials. Fig 3 presents a graphical representation of the retrospective ARIMA, GLARMA and RF models and shows that, overall, the models can successfully detect increases and decreases in the number of submissions and positive submissions, with the exception of the number of weekly positive submissions. The predictive accuracy of the models is summarized in Table 1 and S2 Table. The RMSE and NRMSE in the retrospective analysis vary among the models from 0.966 to 4.429 (Table 1) and 0.111 to 0.210 (S2 Table), respectively, where the large values relate to the monthly submission prediction.

**Table 1:** Predictive accuracy evaluated via the root mean square error (RMSE) of autoregressive integrated moving average (ARIMA), generalized linear autoregressive moving average (GLARMA), and random forest (RF) time series models.

Counts	RMSE retrospective			RMSE prospective		
	ARIMA	GLARMA	RF	ARIMA	GLARMA	RF
Weekly submissions	1.449	1.804	1.822	1.970	1.946	1.890
Monthly submissions	3.823	3.443	4.429	4.926	5.169	4.851
Weekly positive submissions	0.983	0.966	1.010	1.021	1.198	1.018
Monthly positive submissions	1.444	1.759	2.313	2.677	2.786	2.529

In the GLARMA model retrospective analysis, the likelihood ratio test and Wald tests indicate that the GLARMA model provides a better fit than the generalized linear model. Based on the estimated value of the shape parameter, the monthly count time series were modeled with a Poisson GLARMA, and the weekly count time series with a negative binomial GLARMA. The diagnostic plots of the probability integral transformation indicate that the models with the chosen serial correlations are adequate. The results from the analyses reveal a significant upward trend in the number of weekly and monthly positive virological submissions in the study period (*P* < 0.01, *P* < 0.01, respectively); however, this upward trend is not significant (*P* = 0.22 and *P* = 0.12, respectively) in the number of diagnostic submissions at weekly and monthly intervals. Relative to the baseline summer season, the winter, spring, and fall season regression terms were all found to be highly significant (*P* = 0.003, *P* = 0.0004, *P* = 0.001, respectively), indicating that season has a significant impact on the number of weekly diagnostic submissions. Only the fall season was significant (*P* = 0.0419) for the number of weekly positive virological submissions. Furthermore, relative to the August baseline, late fall months, early winter months, spring months and June were found to have a significant impact on the number of monthly diagnostic submissions (*P* < 0.05). Fall and early winter months and May were found significant (*P* < 0.05) when modeling the number of monthly positive virological submissions.

With the retrospective regression RF model, we examined the relative influence of the explanatory variables on the count time series and present the results in Table 2. The importance values for season were found to be among the highest for the monthly and weekly submission counts, but not for the monthly and weekly positive submissions. Trend was found to have the largest importance value for the monthly and weekly positive submission counts. This may suggest that (as was found under the GLARMA model) season affects the monthly and weekly count time series of diagnostic submissions, and the upward trend affects the monthly and weekly count time series of positive diagnostic submissions.

**Table 2:** Retrospective random forest variable importance measurements.

Variables	Percent increase in root mean square error (RMSE)			
	Submission counts		Positive submission counts	
	Monthly	Weekly	Monthly	Weekly
Counts Lag 1	0.41	4.04	1.34	5.48
Counts Lag 2	0.40	2.31	0.19	9.34
Counts Lag 3	4.40	1.50	3.96	0.41
Counts Lag 4	3.26	0.91	2.72	5.87
Counts Lag 5	2.58	0.93	6.05	0.79
Season	5.79	9.61	0.37	3.82
Trend	5.93	3.45	19.21	13.07

### Simulated prospective ARIMA, GLARMA, and RF

**Fig 4:**
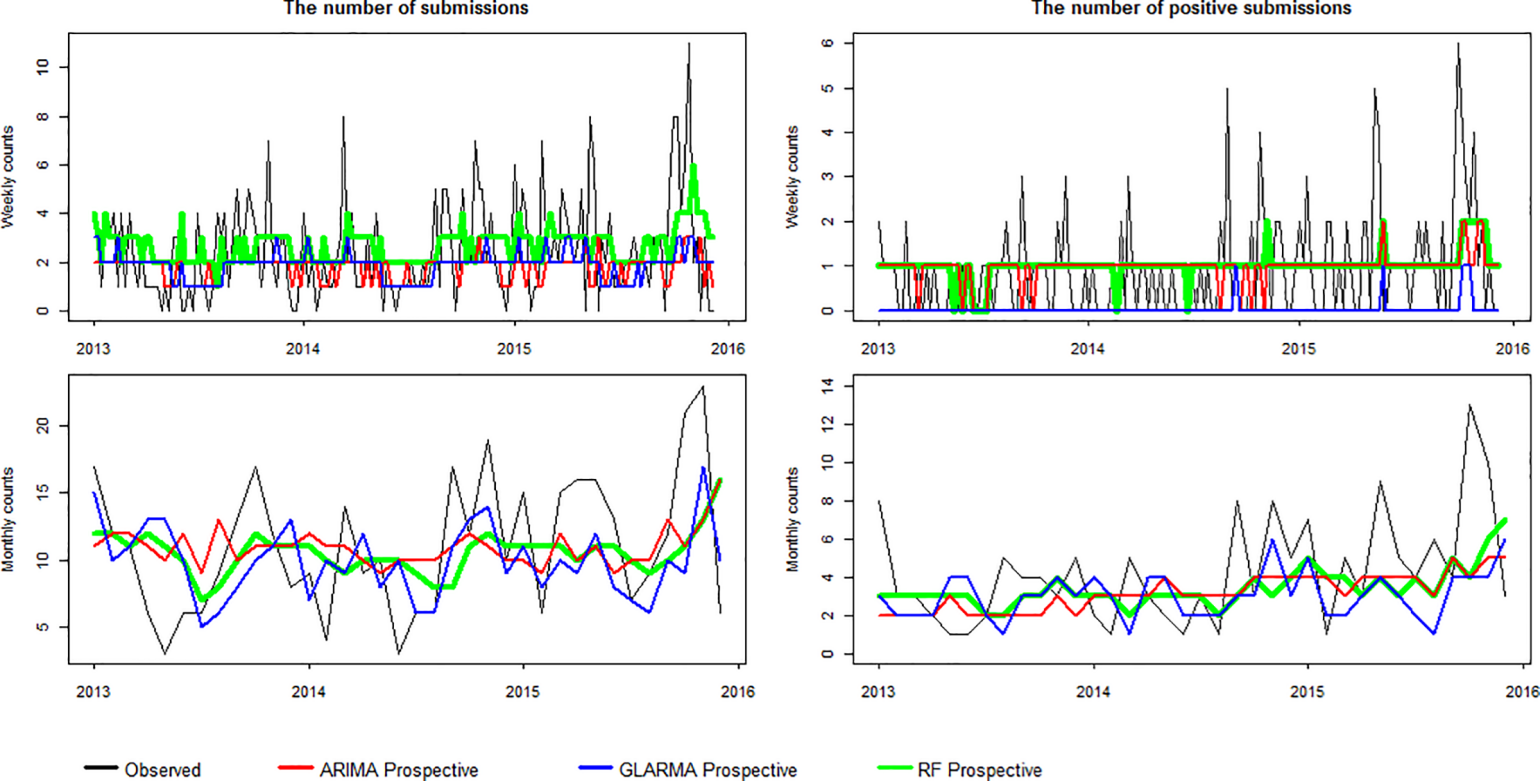
Prospective simulated counts of weekly and monthly submissions and positive submissions for IAV. Counts were predicted for the last three years. The autoregressive integrated moving average (ARIMA) is shown in red, the generalized linear autoregressive moving average (GLARMA) in blue, and the random forest (RF) in green. The actual observations are represented in black.

**Fig 5:**
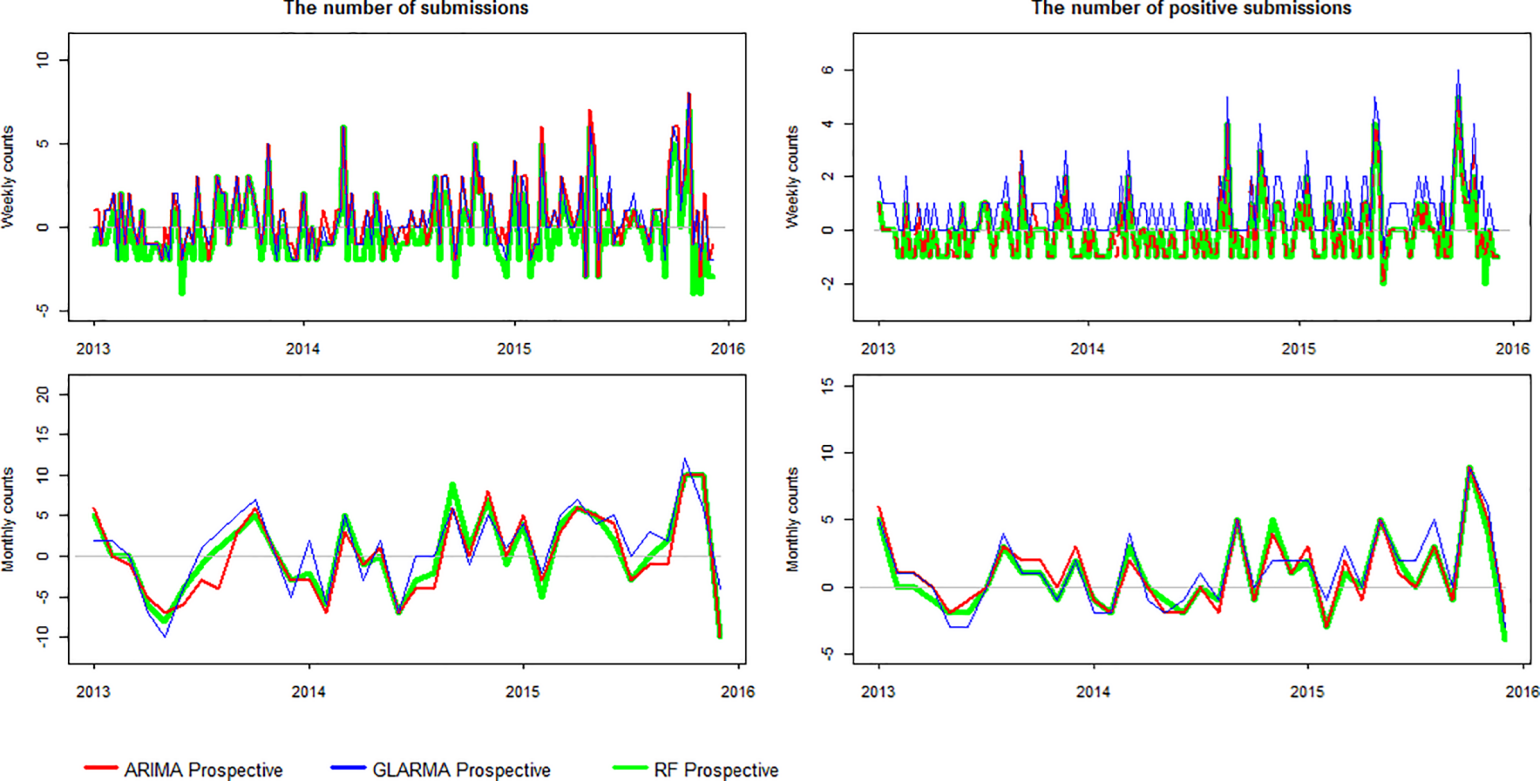
Residuals plots. The residuals were obtained after fitting with simulated prospective autoregressive integrated moving average (ARIMA), generalized linear autoregressive moving average (GLARMA), and random forest (RF) model predicted counts at weekly and monthly intervals.

The predictive accuracy of the simulated prospective time series models are shown in Table 1 and S2 Table. Overall, the predicted and actual counts are very close. The RMSE ranges from 1.018 to 5.1694 with the smallest value for each of the four time series corresponding to the RF model (Table 1). The NRMSE ranges from 0.169 to 0.246 and the smallest value for each of the four time series likewise corresponds to the RF model (S2 Table). Fig 4 illustrates predictions with the simulated prospective models for the last three years. The plots show that high and low counts are not well predicted. Fig 5 contains the residual plots. Figs 4 and 5 both indicate that, on average for the four historical time series, the GLARMA and ARIMA models tend to underestimate counts, whereas the RF model has a tendency to overestimate them. This can be most clearly seen for the weekly data.

The RMSE and the NRMSE for the naïve forecasts of the number of submissions and positive submissions are reported in S3 Table. In all cases but in one (the monthly submissions for the GLARMA model), the predictions with the simple method were less accurate than the predictions obtained under the three time series models.

The predictive accuracy of the simulated prospective models based on the LOSO cross-validation are summarized in S4 Table. Overall, the results are similar to those reported in Table 1 and S2 Table, and the magnitude of the difference between the two validation processes is very small (Table 1 and S2 and S4 Tables).

The simulated prospective model validation results are provided in Table 3 and S5–S15 Tables. Overall, the accuracy of correctly identifying increases and decreases in the number of diagnostic submissions, and positive virological submissions at weekly and monthly intervals, is over 50% (Table 3 and S5–S7 Tables). However, overall the RF model outperformed the GLARMA and ARIMA models. The proportion of increases and decreases correctly identified by the RF model ranged from 61% to 68% (see Supplementary Material (S5–S7 Tables)). Furthermore, the RF tends to predict the actual increases with a higher degree of sensitivity than the GLARMA and ARIMA models, ranging from 0.6 to 0.69.

**Table 3:** Confusion matrix for predicted monthly positive submissions with the prospective autoregressive integrated moving average (ARIMA), generalized linear autoregressive moving average (GLARMA) and random forest (RF) time series models.

Predicted	Actual			Accuracy	Sensitivity
		Up	Down		
ARIMA	Up	0.24	0.10	0.66	0.50
	Down	0.24	0.42		
GLARMA	Up	0.22	0.12	0.63	0.47
	Down	0.25	0.41		
RF	Up	0.27	0.14	0.68	0.60
	Down	0.18	0.41		

The prospective validation results for the seasonal-naïve method are presented in S8–S11 Tables. This method poorly identified the actual increases in the counts, resulting in low accuracy and sensitivity (particularly, for predicting increases in the number of positive virological submissions at weekly and monthly intervals).

The simulated prospective model results based on the LOSO cross-validation are given in S12–S15 Tables. The accuracy with regard to correctly identifying increases and decreases in the number of diagnostic submissions and positive virological submissions at weekly and monthly intervals ranges from 45 to 73%, with the highest and lowest accuracy for each of the four time series corresponding to the RF and ARIMA models, respectively. More specifically, accuracies were 56–73%, 48–59% and 45–62% for the RF, GLARMA and ARIMA models, respectively. Additionally, the proportions of correctly identified increases found were 62–88%, 56–72% and 0–55% for the ARIMA, RF and GLARMA models, respectively.

**Fig 6:**
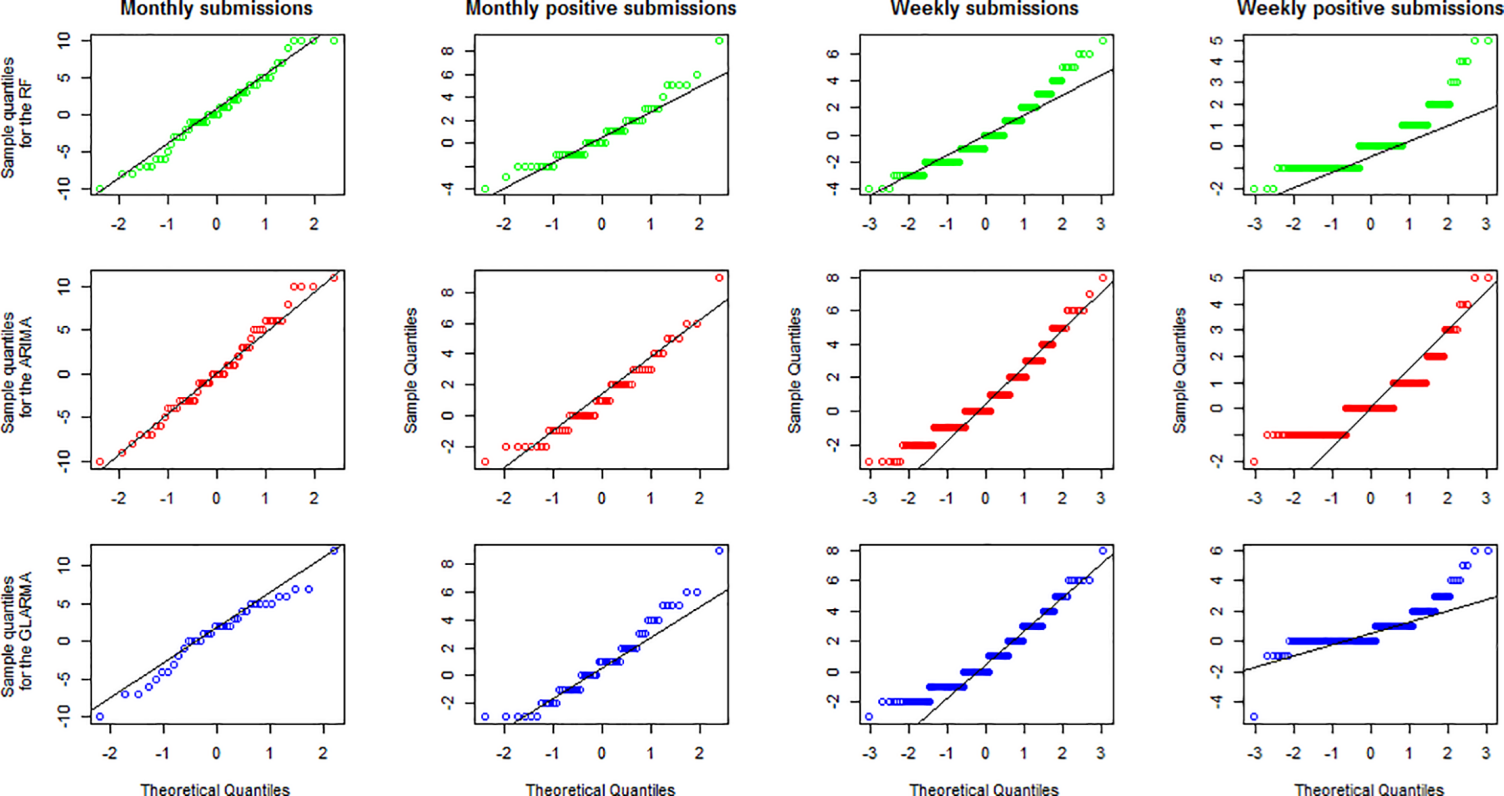
Normal Quantile-|Quantile (Q-Q) plots of the residuals. Residuals were obtained after fitting with simulated prospective autoregressive integrated moving average (ARIMA), generalized linear autoregressive moving average (GLARMA), and random forest (RF) model predicted counts at weekly and monthly intervals.

Fig 6 shows the normal quantile-quantile (QQ) plots of the residuals, plotting the predicted quantiles against the theoretical quantiles. The points for weekly diagnostic submissions, monthly diagnostic submissions, and monthly positive virological submissions seem to fall on a straight line, indicating that the residuals are normally distributed. The QQ plots for the weekly positive time series are roughly linear from -1 to 1 (about 68% of the data), and then the points curve off in the extremities, This suggests the residuals have more extreme values in either the right or left tails than would be expected if they came from a normal distribution. These values correspond to poor prediction of sudden increases or decreases observed.

## Discussion

The literature on different alternatives to analyze time series of IAV or other pathogens in swine populations for surveillance purposes is sparse. To the best of our knowledge, this is the first paper to focus on different approaches for forecasting IAV frequency in swine and to compare the outputs of such approaches to a suitable reference for the purpose of calculating sensitivity and predictability. Based on the AHL swine diagnostic IAV data, we have shown that the prospective RF model outperforms the prospective time series models. A similar conclusion was reached by Kane et al. (2014), who considered the RF and ARIMA models for the prediction of avian influenza outbreaks.

The retrospective and prospective analyses conducted herein highlighted different aspects of modeling performance. Evidence of seasonality could be detected in four time series to various extents. GLARMA models based on the number of submissions per week and per month suggested the existence of seasonal differences among individual months (compared to August) and calendar seasons. Similarly, the RF models based on the latter two time series also indicated season as the most, or second most, important term in the model, respectively. This agrees with a general consensus among experts that respiratory diseases have peaks in the first or second periods of the year [18,19]; it is also consistent with previous results [5]. Evidence for seasonality based on the number of positive virological submissions was less consistent. While several months had statistically higher positive submissions than the month of August for monthly positive submissions, no season other than the fall could be identified as statistically significantly different to summer for weekly positive submissions. In addition, the season effect had among the lowest importance scores among all variables included in RF models. Therefore, strong conclusions about the existence of seasonality based on the number of positive virological submissions cannot be made based on these data alone. However, an increasing trend in the frequency of positive submissions based on monthly and weekly data was detected regardless of the modeling approach considered. This could be a consequence of: (i) improved laboratory methods, or (ii) more effective sampling strategies applied in the field, or (iii) an increasing trend in IAV frequency, or (iv) all, or some, of the above combined.

Prospective ARIMA, GLARMA and RF models were evaluated with respect to their predictive abilities. Results were compared with those obtained with the seasonal-naïve method. Each of the models had problems predicting sudden increases and decreases in the number of diagnostic and positive virological submissions. Such difficulties with forecasting “shocks” are commonly found in economics [20] and outbreak investigations [11,21], among others. An overall comparison of the three time series models indicated that RF models outperformed the ARIMA and GLARMA. The RMSE (or NRMSE) for the prospective forecast of the RF models was the lowest of the three methods. Furthermore, RF models were found to have a tendency to predict increases in counts with a higher degree of sensitivity than ARIMA and GLARMA models. This could be due to several reasons. For instance, in the prospective forecasts the explanatory variables for the GLARMA models remained the same when the model was retrained from one iteration to the next, as well as across the four different time series. However, the parameters of ARIMA and the explanatory variables for RF changed. In fact, within the RF algorithm predictors were sampled at each node of a tree when the model was retrained from one iteration to the next. These adaptive advantages might in part explain the lower RMSE (or NRMSE) of RF over ARIMA and GLARMA, and the lower RMSE (or NRMSE) of ARIMA over GLARMA, in prospective forecasts. Another reason might be that a Box transformation of counts in the ARIMA models did not approximate a Gaussian distribution very well, leading to poor predictive performance. We note that the RF model does not rely on any distributional assumptions and that, although it seems to have higher sensitivity than the other two methods, this model appears to be poorer at predicting decreases in submissions. A possible explanation for not detecting increases as well as decreases could be the existence of factors associated with the variation of the counts that were not included in the analyses.

The three models tested outperformed the seasonal-naïve method. In particular, the predictive values found under the RF model were more accurate and detected the actual increases with a much higher degree of sensitivity than the naïve method. This could be explained by the fact that the seasonal-naïve method ignored all predictor information.

The ARIMA, GLARMA and RF models’ predictive abilities were assessed under the LOSO cross-validation. The RF model was more accurate in predicting the number of diagnostic submissions, and the ARIMA model was more accurate in identifying the actual increases in the positive counts. We note that the three models performed better on the weekly than on the monthly time series. The reason could be that the weekly data had more observations.

In this study, the predictive accuracy of ARIMA, GLARMA and RF models was assessed under different cross-validation approaches. All of the methods yielded qualitatively similar conclusions. However, the application of the validation techniques to time series forecasting was found to not be straightforward due to the inherit serial correlation and non-stationary nature of the data. Therefore, the LOSO cross-validation was used for all three models and the 10-fold cross-validation was used for RF models as part of Breiman’s RF algorithm [9]. It should be noted that both cross-validation techniques are based on partitioning data into training and test sets to estimate the expected prediction error. It is feasible to apply both techniques to RF models, and in both approaches RF models tended to predict increases with a higher degree of sensitivity than ARIMA and GLARMA models. On the other hand, GLARMA models under the LOSO cross-validation failed to predict increases in the number of monthly positive virological submissions. Failure of this cross-validation technique in a time series context was also demonstrated in a study by Moreno-Torres et al. [22] among others. Furthermore, it was found to be difficult to 10-fold cross-validation for GLARMA models because of the need to select appropriate lags for the ARMA components. That is, the algorithm for a GLARMA model is designed in such a way that the use of the 10-fold cross-validation requires the manual specification of the degree of serial dependence for each training set. Moreover, the misspecification of lag structure leads to identifiability issues and lack of convergence of the likelihood optimization algorithm. These were found to result in a very time-consuming validation procedure. There was also a problem with the application of the 10-fold cross-validation for ARIMA models on the weekly time series. The validation process was computationally exhaustive because ARIMA models are fully iterative and have computationally intensive fitting.

Considering the above, it appears that overall the RF is the most accurate model among the ones tested. Based on these findings, and on the fact that detecting increases in disease frequency (sensitivity) is important for veterinary authorities, public health planners and policy makers [23,24], we conclude that the RF models could potentially be used for predicting weekly and monthly counts of IAV submissions, under the conditions that were considered in this analysis. Certainly among the three considered approaches, RF models appear to be the most suitable choice for ongoing reporting systems, and they appear particular suited for predicting increases in disease frequency.

One limitation of this study was the surveillance nature of the data. The rate of swine submissions is likely associated with numerous and varied reasons affecting voluntary participation. The development of innovative strategies to promote participation in a surveillance program would be beneficial for both the swine industry, and due to the pandemic potential of novel influenza virus infections, human populations. Another limitation was that our analyses did not include a number of variables that might be associated with IAV frequency. So, the inclusion of environmental factors (e.g., temperature, humidity) in surveillance models for IAV in swine populations might be another area for further investigation.

## Conclusion

The results from the simulated prospective analysis suggested the RF approach tends to predict increases in the number of diagnostic and positive virological submissions at weekly and monthly intervals with a higher degree of sensitivity than ARIMA and GLARMA models. The predictive performance of each prospective modeling approach was evaluated with the RMSE and NRMSE, which were found to be smallest for the RF model. Overall, the RF modeling approach offers enhanced prediction ability over ARIMA and GLARMA time series models for the diagnostic data under the conditions considered in the analysis of this study. The retrospective ARIMA, GLARMA, and RF models indicate that the fall months and January have the most significant impact on the (increasing) number of weekly and monthly diagnostic and positive virological submissions for IAV infections in Ontario swine populations. A significant linear increasing trend was found for the positive counts at both weekly and monthly intervals. Future research should explore formulations of time series with other factors that could influence the frequency of IAV in swine populations.

## Supporting information

S1 Table Results of the fitted retrospective autoregressive integrated moving average time series model. (PDF)

S2 Table Predictive accuracy via the normalized root mean square error (NRMSE). Predictive accuracy was evaluated for the autoregressive integrated moving average (ARIMA), generalized linear autoregressive moving average (GLARMA), and random forest (RF) time series models.
(PDF)

S3 Table Predictive accuracy for the seasonal-naïve method. Predictive accuracy was evaluated via the root mean square error (RMSE) and the normalized root mean square error (NRMSE).
(PDF)

S4 Table Predictive accuracy using leave-one-season-out cross-validation. Predictive accuracy was evaluated via the root mean square error (RMSE) and the normalized root mean square error (NRMSE).
(PDF)

S5 Table Confusion matrix for predicted monthly diagnostic submissions. Counts were predicted with the prospective autoregressive integrated moving average generalized linear (ARIMA), generalized linear autoregressive moving average (GLARMA), and random forest (RF) time series models.
(PDF)

S6 Table Confusion matrix for predicted weekly diagnostic submissions. Counts were predicted with the prospective autoregressive integrated moving average (ARIMA), generalized linear autoregressive moving average (GLARMA), and random forest (RF) time series models.
(PDF)

S7 Table Confusion matrix for predicted weekly positive submissions. Counts were predicted with the prospective autoregressive integrated moving average (ARIMA), generalized linear autoregressive moving average (GLARMA), and random forest (RF) time series models.
(PDF)

S8 Table Confusion matrix for predicted monthly diagnostic submissions. Counts were predicted with the seasonal-naïve method.
(PDF)

S9 Table Confusion matrix for predicted weekly diagnostic submissions. Counts were predicted with the seasonal-naïve method.
(PDF)

S10 Table Confusion matrix for predicted monthly positive submissions. Counts were predicted with the seasonal-naïve method.
(PDF)

S11 Table Confusion matrix for predicted weekly positive submissions. Counts were predicted with the seasonal-naïve method.
(PDF)

S12 Table Confusion matrix for predicted monthly diagnostic submissions. Counts were predicted with the prospective autoregressive integrated moving average generalized linear (ARIMA), generalized linear autoregressive moving average (GLARMA), and random forest (RF) time series models based on the **leave-one-season-out cross-validation**.
(PDF)

S13 Table Confusion matrix for predicted weekly diagnostic submissions. Counts were predicted with the prospective autoregressive integrated moving average (ARIMA), generalized linear autoregressive moving average (GLARMA), and random forest (RF) time series models **leave-one-season-out cross-validation**.
(PDF)

S14 Table Confusion matrix for predicted monthly positive submissions. Counts were predicted with the prospective autoregressive integrated moving average (ARIMA), generalized linear autoregressive moving average (GLARMA), and random forest (RF) time series models **leave-one-season-out cross-validation**.
(PDF)

S15 Table Confusion matrix for predicted weekly positive submissions. Counts were predicted with the prospective autoregressive integrated moving average (ARIMA), generalized linear autoregressive moving average (GLARMA), and random forest (RF) time series models **leave-one-season-out cross-validation**.
(PDF)

S1 File Enhanced description of material and methods used. (DOC)
