# Supplementary material for: Assessment of autoregressive integrated moving average (ARIMA), generalized linear autoregressive moving average (GLARMA), and random forest (RF) time series regression models for predicting influenza A virus frequency in swine in Ontario, Canada
Source: PLoS One. 2018 Jun 1;13(6):e0198313. doi: 10.1371/journal.pone.0198313 (PMC5983852; doi:10.1371/journal.pone.0198313)
Supplement: S1 Table — (PDF) [file pone.0198313.s001.pdf]

	Coefficient estimates (Standard errors) (p-value)
Monthly diagnostic submissions	MA1 = -0.9136 (0.3397) (0.0084); MA2 = 0.1682 (0.2028) (0.4089); MA3 = -0.2193 (0.1742) (0.2111); MA4 = 0.0249 (0.2312) (0.9145); MA5 = -0.0894 (0.1783) (0.6172); MA6 = -0.0461 (0.1808) (0.7993); MA7 = 0.0670 (0.1857) (0.7190); MA8 = -0.5531 (0.2556) (0.0330); MA9 = -0.0056 (0.2323) (0.9808); MA10 = 0.6050 (0.2255) (0.0086)
Monthly virological positive submissions	MA1 = -0.8172 (< 0.0001); MA2 = -0.1796 (0.1296) (0.1689); MA3 = -0.1536 (0.1100) (0.1657); MA4 = 0.2473 (0.1010) (0.0161)
Weekly diagnostic submissions	AR1 = 0.6941 (0.1745) (< 0.0001); MA1 = -0.5910 (0.1946) (0.0025); SMA1 = 0.1378 (0.0510) (0.0071)
Weekly virological positive submissions	AR1 = 0.1016 (0.0476) (0.0333); AR2 = 0.1286 (0.0477) (0.0073); MA1 = -0.9797(0.0091)(< 0.001)
