# Supplementary material for: Assessment of autoregressive integrated moving average (ARIMA), generalized linear autoregressive moving average (GLARMA), and random forest (RF) time series regression models for predicting influenza A virus frequency in swine in Ontario, Canada
Source: PLoS One. 2018 Jun 1;13(6):e0198313. doi: 10.1371/journal.pone.0198313 (PMC5983852; doi:10.1371/journal.pone.0198313)
Supplement: S2 Table — Predictive accuracy was evaluated for the autoregressive integrated moving average (ARIMA), generalized linear autoregressive moving average (GLARMA), and random forest (RF) time series models. (PDF) [file pone.0198313.s002.pdf]

Counts	NRMSE retrospective			NRMSE prospective		
	**ARIMA**	**GLARMA**	**RF**	**ARIMA**	**GLARMA**	**RF**
Weekly submissions	0.132	0.164	0.166	0.179	0.177	0.172
Monthly submissions	0.182	0.164	0.210	0.235	0.246	0.231
Weekly positive submissions	0.163	0.161	0.168	0.170	0.199	0.169
Monthly positive submissions	0.111	0.135	0.178	0.206	0.214	0.195
