# Supplementary material for: Assessment of autoregressive integrated moving average (ARIMA), generalized linear autoregressive moving average (GLARMA), and random forest (RF) time series regression models for predicting influenza A virus frequency in swine in Ontario, Canada
Source: PLoS One. 2018 Jun 1;13(6):e0198313. doi: 10.1371/journal.pone.0198313 (PMC5983852; doi:10.1371/journal.pone.0198313)
Supplement: S3 Table — Predictive accuracy was evaluated via the root mean square error (RMSE) and the normalized root mean square error (NRMSE). (PDF) [file pone.0198313.s003.pdf]

Counts	RMSE prospective	NRMSE prospective
Weekly submissions	2.125	0.193
Monthly submissions	5.056	0.241
Weekly positive submissions	1.328	0.221
Monthly positive submissions	3.101	0.239
