# Supplementary material for: Assessment of autoregressive integrated moving average (ARIMA), generalized linear autoregressive moving average (GLARMA), and random forest (RF) time series regression models for predicting influenza A virus frequency in swine in Ontario, Canada
Source: PLoS One. 2018 Jun 1;13(6):e0198313. doi: 10.1371/journal.pone.0198313 (PMC5983852; doi:10.1371/journal.pone.0198313)
Supplement: S4 Table — Predictive accuracy was evaluated via the root mean square error (RMSE) and the normalized root mean square error (NRMSE). (PDF) [file pone.0198313.s004.pdf]

Counts	RMSE prospective			NRMSE prospective		
	**ARIMA**	**GLARMA**	**RF**	**ARIMA**	**GLARMA**	**RF**
Weekly submissions	1.851	1.860	1.690	0.168	0.169	0.154
Monthly submissions	4.715	6.031	4.451	0.225	0.287	0.212
Weekly positive submissions	1.013	1.066	0.914	0.169	0.178	0.152
Monthly positive submissions	3.807	3.227	2.342	0.293	0.248	0.180
