# Supplementary material for: Assessment of autoregressive integrated moving average (ARIMA), generalized linear autoregressive moving average (GLARMA), and random forest (RF) time series regression models for predicting influenza A virus frequency in swine in Ontario, Canada
Source: PLoS One. 2018 Jun 1;13(6):e0198313. doi: 10.1371/journal.pone.0198313 (PMC5983852; doi:10.1371/journal.pone.0198313)
Supplement: S6 Table — Counts were predicted with the prospective autoregressive integrated moving average (ARIMA), generalized linear autoregressive moving average (GLARMA), and random forest (RF) time series models. (PDF) [file pone.0198313.s006.pdf]

**Predicted**		**Actual**		**Accuracy**	**Sensitivity**
		**Up**	**Down**		
ARIMA	Up	0.22	0.17	0.59	0.48
	Down	0.24	0.37		
GLARMA	Up	0.20	0.14	0.60	0.44
	Down	0.26	0.40		
RF	Up	0.31	0.25	0.61	0.69
	Down	0.14	0.30		
