# Supplementary material for: Assessment of autoregressive integrated moving average (ARIMA), generalized linear autoregressive moving average (GLARMA), and random forest (RF) time series regression models for predicting influenza A virus frequency in swine in Ontario, Canada
Source: PLoS One. 2018 Jun 1;13(6):e0198313. doi: 10.1371/journal.pone.0198313 (PMC5983852; doi:10.1371/journal.pone.0198313)
Supplement: S8 Table — Counts were predicted with the seasonal-naïve method. (PDF) [file pone.0198313.s008.pdf]

**Predicted**		**Actual**		**Accuracy**	**Sensitivity**
		**Up**	**Down**		
Seasonal naïve method	Up	0.28	0.14	0.63	0.55
	Down	0.23	0.35		
