# Supplementary material for: Assessment of autoregressive integrated moving average (ARIMA), generalized linear autoregressive moving average (GLARMA), and random forest (RF) time series regression models for predicting influenza A virus frequency in swine in Ontario, Canada
Source: PLoS One. 2018 Jun 1;13(6):e0198313. doi: 10.1371/journal.pone.0198313 (PMC5983852; doi:10.1371/journal.pone.0198313)
Supplement: S10 Table — Counts were predicted with the seasonal-naïve method. (PDF) [file pone.0198313.s010.pdf]

Predicted		Actual		Accuracy	Sensitivity
		**Up**	**Down**		
Seasonal naïve method	Up	0.13	0.07	0.59	0.28
	Down	0.34	0.46		
