# Supplementary material for: Assessment of autoregressive integrated moving average (ARIMA), generalized linear autoregressive moving average (GLARMA), and random forest (RF) time series regression models for predicting influenza A virus frequency in swine in Ontario, Canada
Source: PLoS One. 2018 Jun 1;13(6):e0198313. doi: 10.1371/journal.pone.0198313 (PMC5983852; doi:10.1371/journal.pone.0198313)
Supplement: S11 Table — Counts were predicted with the seasonal-naïve method. (PDF) [file pone.0198313.s011.pdf]

**Predicted**		**Actual**		**Accuracy**	**Sensitivity**
		**Up**	**Down**		
Seasonal naïve method	Up	0.03	0.05	0.56	0.07
	Down	0.39	0.53		
