# Supplementary material for: Assessment of autoregressive integrated moving average (ARIMA), generalized linear autoregressive moving average (GLARMA), and random forest (RF) time series regression models for predicting influenza A virus frequency in swine in Ontario, Canada
Source: PLoS One. 2018 Jun 1;13(6):e0198313. doi: 10.1371/journal.pone.0198313 (PMC5983852; doi:10.1371/journal.pone.0198313)
Supplement: S13 Table — Counts were predicted with the prospective autoregressive integrated moving average (ARIMA), generalized linear autoregressive moving average (GLARMA), and random forest (RF) time series models leave-one-season-out cross-validation. (PDF) [file pone.0198313.s013.pdf]

| **Predicted** | | **Actual** | | **Accuracy** | **Sensitivity** |
|---|---|---|---|---|---|
| | | **Up** | **Down** | | |
| ARIMA | Up | 0.28 | 0.26 | 0.57 | 0.62 |
| | Down | 0.17 | 0.29 | | |
| GLARMA | Up | 0.26 | 0.22 | 0.57 | 0.55 |
| | Down | 0.21 | 0.31 | | |
| RF | Up | 0.33 | 0.14 | 0.73 | 0.72 |
| | Down | 0.13 | 0.40 | | |
