# Supplementary material for: Assessment of autoregressive integrated moving average (ARIMA), generalized linear autoregressive moving average (GLARMA), and random forest (RF) time series regression models for predicting influenza A virus frequency in swine in Ontario, Canada
Source: PLoS One. 2018 Jun 1;13(6):e0198313. doi: 10.1371/journal.pone.0198313 (PMC5983852; doi:10.1371/journal.pone.0198313)
Supplement: S14 Table — Counts were predicted with the prospective autoregressive integrated moving average (ARIMA), generalized linear autoregressive moving average (GLARMA), and random forest (RF) time series models leave-one-season-out cross-validation. (PDF) [file pone.0198313.s014.pdf]

Predicted		Actual		Accuracy	Sensitivity
		Up	Down		
ARIMA	Up	0.36	0.45	0.50	0.88
	Down	0.05	0.14		
GLARMA	Up	0.00	0.02	0.48	0.00
	Down	0.50	0.48		
RF	Up	0.27	0.25	0.56	0.58
	Down	0.19	0.29		
