# Supplementary material for: Assessment of autoregressive integrated moving average (ARIMA), generalized linear autoregressive moving average (GLARMA), and random forest (RF) time series regression models for predicting influenza A virus frequency in swine in Ontario, Canada
Source: PLoS One. 2018 Jun 1;13(6):e0198313. doi: 10.1371/journal.pone.0198313 (PMC5983852; doi:10.1371/journal.pone.0198313)
Supplement: S15 Table — Counts were predicted with the prospective autoregressive integrated moving average (ARIMA), generalized linear autoregressive moving average (GLARMA), and random forest (RF) time series models leave-one-season-out cross-validation. (PDF) [file pone.0198313.s015.pdf]

**Predicted**		**Actual**		**Accuracy**	**Sensitivity**
		**Up**	**Down**		
ARIMA	Up	0.24	0.49	0.45	0.80
	Down	0.06	0.21		
GLARMA	Up	0.08	0.13	0.59	0.22
	Down	0.28	0.51		
RF	Up	0.19	0.13	0.72	0.56
	Down	0.15	0.53		
