# Supplementary material for: Assessment of autoregressive integrated moving average (ARIMA), generalized linear autoregressive moving average (GLARMA), and random forest (RF) time series regression models for predicting influenza A virus frequency in swine in Ontario, Canada
Source: PLoS One. 2018 Jun 1;13(6):e0198313. doi: 10.1371/journal.pone.0198313 (PMC5983852; doi:10.1371/journal.pone.0198313)
Supplement: S1 File — (DOC) [file pone.0198313.s016.doc]

In this section, the modeling approaches are described in greater detail. Particularly, this section provides explanations of theory of (1) an Autoregressive Integrated Moving Average model (ARIMA), (2) a Generalized Linear Autoregressive Moving Average model (GLARMA), (3) a Random Forest model (RF), (4) the root mean square error (RMSE), (5) the normalized root mean square error (NRMSE), and the seasonal naïve method.

**ARIMA**

We first considered the representation of the observed time series via an ARIMA model (cite). These models consist of three components: an autoregressive (AR) process, a moving average (MA) process, and the integrated (I) part.

Let
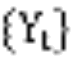
 be an arbitrary time series, a sequence of measurements of the same variable collected over time. Let
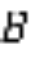
 be the lag operator that shifts
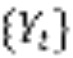
 back
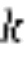
 periods, also known as a backshift operator, defined as
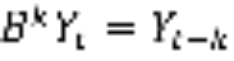
. Then, in an AR process
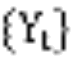
 of order *p* is defined as a linear aggregate of its *p* predecessors and a stochastic term
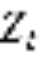
 and can be written as


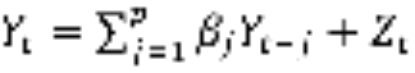
,

where
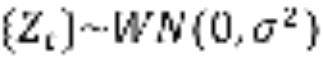
 and
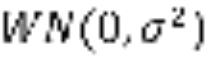
 is white noise with variance given by
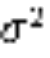
, and the
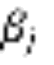
are weights. A moving average process of order *q* is a linear filter applied to current and *q* past stochastic terms
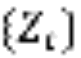
 and is defined as


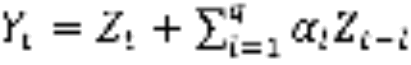
,

where
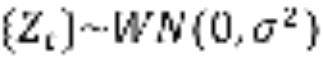
, and the
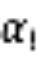
are weights. In terms of the lag operator
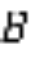
, an AR process of order
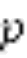
, AR(
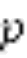
), and a MA process of order
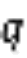
, MA(
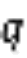
), can be expressed as


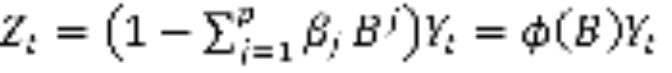
 and
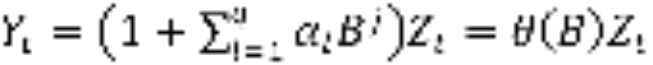
, respectively, where
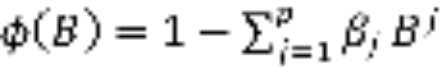
 and
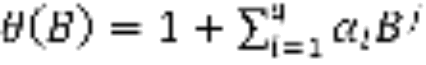
 are the polynomials of degree
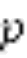
 and
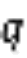
. The integrated part transforms
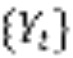
 to account for non-stationary, i.e. to eliminate the effect of the trend on the value of a time series at different times. The transformation consists of taking the differences between lagged series observations. The first lag difference of
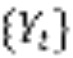
 is defined by the expression
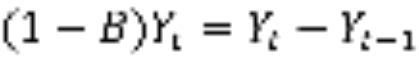
. High order differences are defined by repeated application. That is, the second difference can be written as
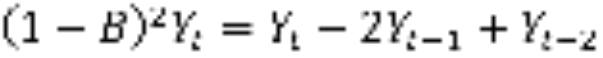
, and the
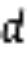
 difference can be defined by expression
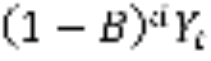
. Combining the three terms together, it can be said that a time series
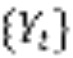
 is an ARIMA process of order
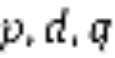
,
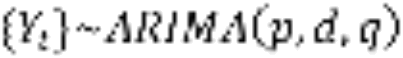
, with
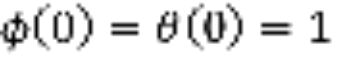
 and it can be expressed as


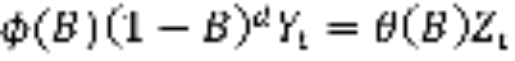
,

where after the
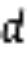
th difference of
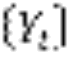
, the time series is a stationary ARMA process of order
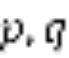
;
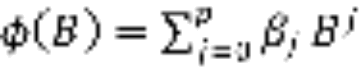
 and
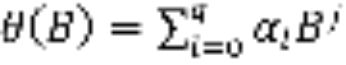
;
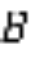
 is a backshift operator. For more information, the reader is referred to [1,2].

**GLARMA**

The general form of the GLARMA is defined as


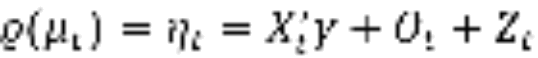
,

where
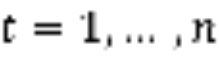
;
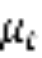
 is the expected value of a sequence of counts
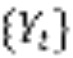
 given the previous information set
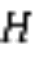
,
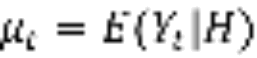
 and
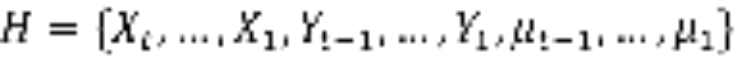
, and it is related to the link function
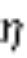
 by a twice-differentiable one-to-one monotonic function
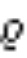
,
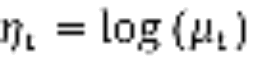
 for the
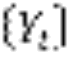
 that follows either a Poisson distribution or a Negative Binomial distribution and


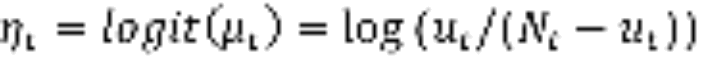
 for the
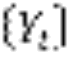
 that follows a Binomial distribution;
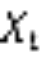
 is the
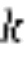
-dimensional vector of time-dependent explanatory variables with the corresponding regression coefficients
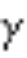
;
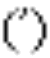
 is transpose;
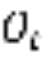
 is the varying population at risk or sample size;
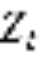
 is the residuals that follow an
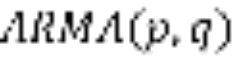
 process and defined as
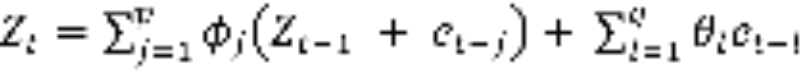
 where
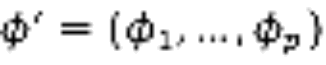
 and
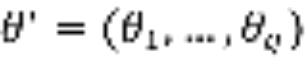
 are the AR and MA parameters, respectively;
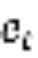
is the predictive residuals,
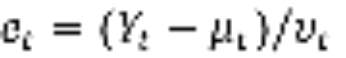
where
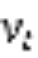
 is some scaling sequence [3]. The GLARMA models are explained in considerably more detail in [3–5].

**RF**

Breiman's RF algorithm [6] builds an ensemble of tree predictors such that each tree with branches and nodes is independently constructed from a different bootstrap sample, based on the principle of recursive partitioning where data space is recursively partitioned into smaller groups to minimize the amount of variation in each subspace. To minimize the chance of having trees with similar structures and thereby reducing the correlation among predictors while maintaining strength, Breiman applied two layers of randomness. He used the bootstrapping technique to construct trees and implemented random split selection where at each node the best split is selected from the
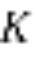
 randomly chosen predictors. The resulting forests are accurate regressors and are relatively robust to outliers and noise.

The regression with the RF starts with the generation of a training set for each tree by drawing a bootstrap sample from the original data. The training sets are used to train and grow trees, while unselected observations referred to as the “out-of-bag” (OOB) samples are used to evaluate the tree predictive performance and to estimate the importance of variables. A tree construction is performed by recursively splitting the data at a node. For each split, a small number
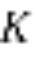
 of the original predictors is sampled. Every different value of every different randomly selected predictor is taken to find the predictor and split value that best reduces the overall sums of squares error. Nodes are further split until a desired tree model stopping criteria is reached. Within the grown trees, if-then statements are used to generate an individual prediction from each tree. The predictions are then averaged to give the forest’s prediction.

The performance of the RF model can be improved by finding an optimal number of randomly selected predictors and number of trees, associated with the smallest estimate of either the cross-validation (CV) error or the OOB error. The CV approach for the RF involves the variable importance measure and is based on random partitioning of the data into
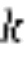
 parts and training a model on each training set and recording predictions on the test set. The CV error is the mean squared error at each variable reduction step computed from the aggregated predictions from all
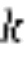
 CV partitions [7]. The OOB error estimate is the mean squared error of the aggregated prediction errors obtained using the OOB data and the trees grown within the training set and defined as
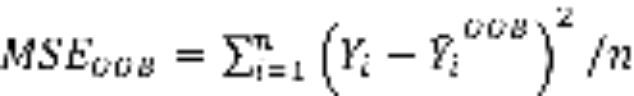
 where  
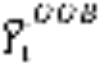
 is the average of the OOB predictions for the
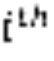
 observation.

The RF also provides a measure of variable importance, which can be useful for the model interpretation. The variable importance measure is obtained based on the aggregated difference in predictive performance between the non-permuted OOB data and the permuted values for each predictor at the time from the OOB data for each tree across the entire forest. The output is the percent increase in the mean squared error (MSE). If a predictor contributes to prediction accuracy, then after injecting random noise (e.g., permuting values of the predictor) the accuracy of the predictor would decrease and the percent increase in the MSE would be larger. If a predictor is irrelevant, noising it would have little effect on the performance and the percent increase in the MSE would be small. The variable importance measure could be used to determine relative influence of a predictor on each historical count time series.

For more detailed explanation of recursive partitioning and the Random Forest procedure, reader is referred to [6,8].

**RMSE**

The root mean square error (RMSE) represents the sample standard deviation of the differences between estimates or predictions and the values actually observed. The RMSE can be written as


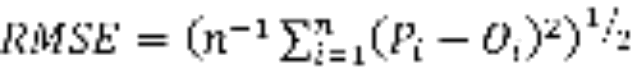
 , (1)

where
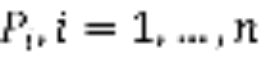
, is the
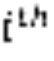
 model estimate or the
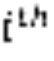
 prediction generated at each iteration;
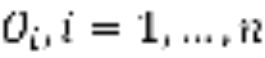
, is the
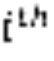
 matched actual observation.

This RMSE definition was used for both retrospective and prospective analyses. The predicted values were generated for each analysis and each modeling approach differently. That is, in the retrospective analysis with the ARIMA model the predicted counts for each time series were produced with the forecast function in R after performing the model estimation procedure on each historical dataset. In the simulated prospective analysis with the ARIMA model, the predicted counts for each time series were generated with the forecast function in R after retraining the model at each iteration using the updated data, excluding the training period.

The forecasts for each time series with the retrospective GLARMA model were extracted after performing the GLARMA modeling process on each historical dataset. The prospective forecast for each time series were generated after retraining the model at each iteration using the updated data and excluding the training period.

In the retrospective analysis with the RF model, the predicted values for each time series were based on the out-of bag samples (please see the description of the RF model above), and the prospective forecast for each time series were generated also based on the out-of- bag samples at each iteration after performing the RF regression using the updated data.

**NRMSE**

Normalizing the root means square error allows to comparing datasets or models with different scales. Although there is no consistent means in the literature, the common choice is to use the range defined as the maximum value,
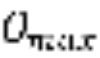
, minus the minimum value,
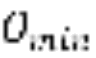
 of the measured data [9]:


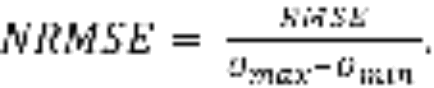
 (2)

This NRMSE definition was used for both retrospective and prospective analyses. The RMSE used in the formula were calculated as described above.

**Seasonal naïve method**

This method was used as benchmark for prospective ARIMA, GLARMA and RF models and was based on weekly (or monthly) averages over past years for each historical time series. That is, each predicted value was set to be equal to the mean of the values from the same week/month of the previous years [10]. The forecasting procedure started from the first week (or month) in January, averaging the values over the three past years. Then, the process proceeded by iteratively adding a successive week (or month) and predicting the number of submissions or positive submissions. The forecast obtained with this method were used to compute RMSE, accuracy and sensitivity to compare these values with those obtained with the ARIMA, GLARMA and RF models.

**References**

1. Brockwell P, Davis R. Time series: theory and methods [Internet]. 2013. Available: https://books.google.ca/books?hl=en&lr=&id=DJ_lBwAAQBAJ&oi=fnd&pg=PR7&ots=AbwFfE-EiV&sig=ztLrZ2_A4Gb_-KS9e7gwZvbk7W8

2. Diggle P. Time series; a biostatistical introduction [Internet]. 1990. Available: http://www.sidalc.net/cgi-bin/wxis.exe/?IsisScript=COLPOS.xis&method=post&formato=2&cantidad=1&expresion=mfn=008734

3. Dunsmuir W, Li G, Scott D. glarma: Generalized Linear Autoregressive Moving Average Models [Internet]. 2015. Available: https://cran.r-project.org/package=glarma

4. Davis R, Dunsmuir W, Wang Y. Modelling Time Series of Count Data [Internet]. STATISTICS TEXTBOOKS AND MONOGRAPHS. MARCEL DEKKER AG; 1999. Available: http://www.stat.columbia.edu/~rdavis/lectures/montreal.pdf

5. Benjamin MA, Rigby RA, Stasinopoulos MD. Generalized Autoregressive Moving Average Models. Source J Am Stat Assoc. 2003;98: 214–223. Available: http://www.jstor.org/stable/30045208

6. Breiman L. Random Forests. Mach Learn. Kluwer Academic Publishers; 2001;45: 5–32. doi:10.1023/A:1010933404324

7. Svetnik V, Liaw A, Tong C, Wang T. Application of Breiman’s Random Forest to Modeling Structure-Activity Relationships of Pharmaceutical Molecules [Internet]. International Workshop on Multiple Classifier Systems. Springer Berlin Heidelberg; 2004. doi:10.1007/978-3-540-25966-4_33

8. Kuhn M, Johnson K. Applied Predictive Modeling. New York: Springer; 2013.

9. Statistics - CIRPwiki [Internet]. [cited 16 Jan 2018]. Available: http://cirpwiki.info/wiki/Statistics#Normalization

10. Hyndman RJ, Athanasopoulos G. Forecasting : principles and practice [Internet]. Available: https://books.google.ca/books?id=gDuRBAAAQBAJ&lr=&source=gbs_navlinks_s
